# Peripheral T-Cell Lymphoma: From Biological Research to New Therapies

**DOI:** 10.3390/cancers15164192

**Published:** 2023-08-21

**Authors:** Shingo Nakahata, Kazuhiro Morishita

**Affiliations:** 1 Division of Tumor and Cellular Biochemistry, Department of Medical Sciences, University of Miyazaki, Miyazaki 889-1692, Japan; 2 Division of HTLV-1/ATL Carcinogenesis and Therapeutics, Joint Research Center for Human Retrovirus Infection, Kagoshima University, Kagoshima 890-8544, Japan; 3 Project for Advanced Medical Research and Development, Project Research Division, Frontier Science Research Center, University of Miyazaki, Miyazaki 889-1692, Japan

This series of six articles (four reviews and two original articles) is presented by international leaders on peripheral T-cell lymphomas (PTCL). PTCL is a heterogeneous group of neoplasms originating from T cells that migrate to peripheral lymphoid tissues after differentiation and maturation in the thymus, and accounts for 10–15% of all non-Hodgkin’s lymphomas (NHL). Compared with B-cell lymphoma, disease-specific molecular markers for PTCL are scarce, and PTCL comprises many different entities. In addition, compared to B-cell lymphoma, established treatment methods for PTCL are limited and the prognosis of most PTCL cases remains poor. This special issue summarizes recent findings on the molecular pathology and potential therapeutic targets of PTCL and presents new candidate biomarkers. In addition, considering virus-associated PTCL, this issue includes a review of the methods for the prevention of human T-cell leukemia virus type 1 (HTLV-1) infection and a genomic analysis of Epstein–Barr virus (EBV).

Adult T-cell leukemia/lymphoma (ATL) is a malignant tumor with poor prognosis caused by HTLV-1 infection. Over 10 million HTLV-1 carriers are estimated worldwide, mainly in southwestern Japan, sub-Saharan Africa, South America, and the Caribbean region, and 2–7% of HTLV-1 carriers develop ATL. The main routes of HTLV-1 infection are mother-to-child transmission through breast milk and horizontal transmission through sexual intercourse and blood transfusion; mother-to-child transmission is known to be involved in the development of ATL. Itabashi and Miyazawa [1] elegantly review the latest findings, which range from the mechanism of mother-to-child transmission of HTLV-1 to epidemiological data and preventive measures. In Japan, all pregnant women are currently tested for HTLV-1 antibodies, and health guidance is provided if positive results are confirmed. A Japanese cohort analysis did not identify any differences in HTLV-1 infection when breastfeeding was restricted to up to 3 months compared to when breastfeeding was completely formula-based; however, the risk of infection increased when the duration of breastfeeding extended to 6 months. Although the mechanism of suppression of HTLV-1 infection during short-term breastfeeding has not yet been elucidated, research suggests that the transfer of maternal antibodies to infants may be involved. Furthermore, this review [1] discusses how mother-to-child transmission is established, highlighting the possibility that infected lymphocytes migrate to the infant’s gastrointestinal tract and that the infant’s tonsils may also be a target. In addition, the heightened risk of all-cause mortality associated with HTLV-1 infection necessitates considerations of issues related to HTLV-1 infection on a global scale.

PTCL with a T follicular helper phenotype (PTCL-TFH), including angioimmunoblastic T-cell lymphoma (AITL), is a major PTCL entity. However, compared to other lymphomas, such as B-cell lymphoma, this subtype is rare, posing challenges in launching clinical trials. As summarized in the review by Krug et al. [2] in this special issue, mutations in genes that encode DNA methylation regulators and T-cell receptor (TCR) signaling molecules, including Tet methylcytosine dioxygenase 2 (TET2), are frequent in PTCL-TFH. TET2 mutations appear to coexist with mutations in the small GTPase Ras homolog family member A (RHOA), and Tet2^−/−^RHOA(G17V) mice exhibit AITL-like pathology. Cyclophosphamide, hydroxydaunorubicin, vincristine, and prednisone (CHOP) chemotherapy is the first-line treatment option in PTCL-TFH, but the response rate is not satisfactory. Clinical trials that used a combination of various molecular target drugs, such as the anti-CD20 monoclonal antibody rituximab and lenalidomide, with CHOP have also yielded unsatisfactory results. A possible next step would be to identify new molecular targets, and Krug et al. [2] delineate potential pathways. In particular, RHOA is involved in the activation of T cells, and, indeed, mutations in genes that constitute TCR signaling pathways occur frequently in AITL. Cyclosporine is an inhibitor of calcineurin, a key enzyme in the TCR pathway, which has shown good responses in clinical trials against AITL. In addition, the possibility of immunotherapy targeting inducible costimulatory molecules (ICOS) and programmed cell death 1 (PD-1) has been summarized. Regarding PD-1, monotherapy has not proven effective in some PTCL cases, for example, in ATL, which develops into malignancy after treatment. Chimeric antigen receptor (CAR)-T cell therapy using CD30 antibodies is also being tested in clinical trials for AITL. Moreover, PTCL and AITL cells are known to acquire unique metabolic signatures and may serve as new targets.

Regarding the clonal evolution of PTCL, genomic mutations commonly appear in molecules involved in the TCR signaling pathway, which Liu et al. [3] focused on and comprehensively summarized in this special issue. Mutations in RHOA and vav guanine nucleotide exchange factor 1 (VAV1) are frequently observed in AITL, PTCL not otherwise specified (PTCL-NOS), and ATL. In addition, mutations in phosphoripase C, gamma 1 (PLCγ1), and caspase recruitment domain family, member 11 (CARD11), downstream of TCR, are frequently noted in PTCL subtypes, including AITL, PTCL-NOS, and ATL, but are rare in anaplastic large-cell lymphoma (ALCL), enteropathy-associated T cell lymphoma (EATL), and extranodal nasal natural killer (NK)/T cell lymphoma (NKTCL). These differences suggest the presence of diverse mechanisms underlying clonal evolution. In addition, mutations and fusion genes of CD28, a T cell activator, are characteristic genetic abnormalities in PTCL. In particular, CTLA4–CD28 fusion (CTLA4 is an immune checkpoint receptor that negatively regulates immune responses) has been detected at a frequency of approximately 40% in AITL, PTCL-NOS, and NKTCL and should be noted as a therapeutic target. Interestingly, genetic changes in TCR signaling and its costimulatory factors occur in almost all PTCL cases. As pointed out by Liu et al. [3], PTCL is a cancer that is difficult to classify and diagnose and advancing a broader genomic analysis of PTCL would be useful for the classification of disease types based on mutations in TCR signaling components and for the development of new therapeutic agents.

On the other hand, Al-Khreisat et al. [4] in this special issue reviewed the relationship between PTCL and genetic abnormalities in Notch receptor 1 (NOTCH1), GATA binding protein 3 (GATA3), and c-MYC, which play important roles in T cell development in the thymus. Previous findings on these genes have mostly been reported in normal T-cell development and T-cell acute lymphoblastic leukemia (T-ALL). NOTCH1 is activated through t(7;9)(q34;34.3) translocation and mutation and regulates cell proliferation and the cell cycle in T-ALL cells. NOTCH1 is known to directly or indirectly upregulate GATA3 and c-MYC expression. The expression of NOTCH1, GATA3, and c-MYC in PTCL has been reported to be associated with patient prognosis. Further analyses will clarify the role of NOTCH1, GATA3, and c-MYC in PTCL and their potential as therapeutic targets.

In addition to HTLV-1 that can cause ATL, EBV is another virus involved in PTCL development. EBV, a herpesvirus that infects most adults and remains latent, is detected in most Burkitt lymphomas and NKTCL. Regarding the carcinogenic mechanisms induced by EBV, the virus is thought to be reactivated by some factor, inducing infected B-cell carcinogenesis, and EBV infection likely promotes tumor development by altering the immune microenvironment. On the other hand, EBV is detected in approximately 90% of AITL cases, and researchers speculate that it is related to AITL pathogenesis. Bahri et al. [5] in this special issue reported a whole-genome analysis of EBV using biopsies from 16 AITL cases. The findings indicate that no EBV strain was specific to AITL and the virus remained in a latent state. However, importantly, an EBV strain with poor prognosis in AITL was identified, suggesting that this virus may be involved in the pathogenesis of AITL. Elucidating the physiological role of EBV in AITL may thus be useful for developing therapeutic strategies.

Stimulator of interferon genes (STING) plays an important role not only in the regulation of activity against viral infection but also in tumor inflammation and antitumor immune responses. Xagoraris et al. [6] in this special issue comprehensively analyzed the expression of STING in PTCL using biopsy tissues and reported that elevated STING expression was observed in 95% of anaplastic lymphoma kinase (ALK)-positive ALCL samples, 62% of ALK-negative ALCL samples, 79% of PTCL-NOS, and 56% of NK/T-nasal type, while STING was not expressed in B-cell NHL samples. Furthermore, in the analyzed NKTCL specimens, a correlation between STING expression and prognosis was not observed. This suggests that STING may be a new biomarker or molecular target for NKTCL. 

The above series of papers suggest that PTCL entities are associated both with common molecular mechanisms and mechanisms characteristic of each subtype. We expect that further analyses will lead to the identification of disease-specific molecular markers and the establishment of treatment methods.
